# State of newborn care in South Sudan’s displacement camps: a descriptive study of facility-based deliveries

**DOI:** 10.1186/s12978-017-0417-z

**Published:** 2017-11-29

**Authors:** Samira Sami, Kate Kerber, Solomon Kenyi, Ribka Amsalu, Barbara Tomczyk, Debra Jackson, Alexander Dimiti, Elaine Scudder, Janet Meyers, Jean Paul De Charles Umurungi, Kemish Kenneth, Luke C. Mullany

**Affiliations:** 1 Department of International Health, Johns Hopkins Bloomberg School of Public Health, 615 N. Wolfe Street, Baltimore, MD USA; 2 Save the Children US, Washington, DC USA; 3 International Medical Corps, Juba, South Sudan; 4 U.S. Centers for Disease Control and Prevention, Atlanta, GA USA; 5 UNICEF, New York, NY USA; 6 Republic of South Sudan Ministry of Health, Juba, South Sudan; 7 UNICEF, Aweil, South Sudan

**Keywords:** Newborn health, South Sudan, Conflict, Health services, Quality of care, Knowledge, Postnatal care, Displaced populations, Newborn commodities

## Abstract

**Background:**

Approximately 2.7 million neonatal deaths occur annually, with highest rates of neonatal mortality in countries that have recently experienced conflict. Constant instability in South Sudan further strains a weakened health system and poses public health challenges during the neonatal period. We aimed to describe the state of newborn facility-level care in displaced person camps across Juba, Malakal, and Maban.

**Methods:**

We conducted clinical observations of the labor and delivery period, exit interviews with recently delivered mothers, health facility assessments, and direct observations of midwife time-use. Study participants were mother-newborn pairs who sought services and birth attendants who provided delivery services between April and June 2016 in five health facilities.

**Results:**

Facilities were found to be lacking the recommended medical supplies for essential newborn care. Two of the five facilities had skilled midwives working during all operating hours, with 6.2% of their time spent on postnatal care. Selected components of thermal care (62.5%), infection prevention (74.8%), and feeding support (63.6%) were commonly practiced, but postnatal monitoring (27.7%) was less consistently observed. Differences were found when comparing the primary care level to the hospital (thermal: relative risk [RR] 0.48 [95% CI] 0.40–0.58; infection: RR 1.28 [1.11–1.47]; feeding: RR 0.49 [0.40–0.58]; postnatal: RR 3.17 [2.01–5.00]). In the primary care level, relative to newborns delivered by traditional birth attendants, those delivered by skilled attendants were more likely to receive postnatal monitoring (RR 1.59 [1.09-2.32]), but other practices were not statistically different. Mothers’ knowledge of danger signs was poor, with fever as the highest reported (44.8%) followed by not feeding well (41.0%), difficulty breathing (28.9%), reduced activity (27.7%), feeling cold (18.0%) and convulsions (11.2%).

**Conclusions:**

Addressing health service delivery in contexts affected by conflict is vital to reducing the global newborn mortality rate and reaching the Sustainable Development Goals. Gaps in intrapartum and postnatal care, particularly skilled care at birth, suggest a critical need to build the capacity of the existing health workforce while increasing access to skilled deliveries.

## Plain English summary

Countries affected by conflict have the highest rates of newborn mortality. South Sudan is one of these settings facing an ongoing humanitarian emergency, which is leading to gaps in the delivery of health care. Our study was designed to provide a description of the quality of health care services received by mothers and newborns in displaced person camps in South Sudan. In the five study health facilities, we assessed the availability of newborn medical supplies and skilled birth attendants. Birth attendants were observed to understand how much of their time was spent on newborn care and to document clinical practices during delivery and immediately after birth. Mothers who delivered in the facility were also interviewed about their knowledge of newborn danger signs. This study found that the health facilities lacked the recommended medical supplies particularly for the care of small and sick babies. It was estimated that more than half of the babies born in the facility received thermal care, infection prevention and feeding support, but monitoring the newborn immediately after delivery was low. Certain practices were found to be lower among newborns delivered in a primary care facility or by an unskilled birth attendant. Mothers had poor knowledge of newborn danger signs such as difficulty breathing, reduced activity and convulsions. Addressing the gaps identified in this study is vital to reducing the global rate of newborn mortality. Improving access to newborn supplies and skilled attendants can be integrated in humanitarian response to provide lifesaving intrapartum and postnatal care.

## Background

Deaths among children under five years have decreased from an estimated 11.9 million to 5.9 million between 1990 and 2015 [1]. As progress in reducing neonatal mortality (i.e. first 28 days of life) has lagged improvement made in the postnatal period, the 2.7 million deaths during the first month now account for 45.1% of all child deaths [1, 2]. While the overwhelming majority of neonatal deaths occur in low- and middle-income countries, regional differences exist. In particular, Sub-Saharan Africa’s neonatal mortality rate (NMR) exceeds 28.6 per 1000 livebirths; this region has experienced the slowest annual reduction rate (1% per year) between 1990 and 2009 [3, 4]. Of the 15 countries with the highest NMR, 14 have experienced a recent humanitarian emergency due to conflict or political instability [5].

As mortality risk peaks during the first 24 h of life [4], it is critical to ensure that delivery attendants can provide safe and timely care to maximize improvements in survival [6, 7]. Complications of prematurity (36%), severe infections (23%) and intrapartum-related conditions resulting in perinatal asphyxia (23%) are the main causes of neonatal death [6, 7]. The 2014 Lancet Neonatal Survival Series identified evidence-based interventions for reducing neonatal mortality. For instance, during the intrapartum period, clean birth practices are estimated to reduce neonatal mortality due to sepsis by 38% in the facility. During the postnatal period, newborn resuscitation (30–38% reduction), early initiation of breastfeeding (44%), and prevention and management of hypothermia (10–20%) have proven to reduce all-cause neonatal mortality [2, 8, 9].

Gaps in the availability and utilization of health services remain a major contributor of high mortality in humanitarian settings [10]. One such setting, South Sudan, has a high maternal mortality ratio (MMR) (789 deaths per 100,000 live births) and NMR (39 deaths per 1000 live births), few births (19%) are attended by skilled health workers, and care for small and sick newborns is limited to hospitals [11, 12]. South Sudan faces a severe shortage of health workers and has a limited number of training institutions [13]. Moreover, weak pharmaceutical and medical supply chains plus inaccessibility due to persistent conflict pose significant challenges to the delivery of quality health services. A recent national health facility survey found only a quarter of surveyed health facilities had the supplies or equipment to provide basic newborn care [14]. Much of the facility-based newborn health services for displaced populations in South Sudan are managed by international organizations through primary health units, primary health care centers (PHCCs) and hospitals [14]. Where services do exist, there are very limited data on the quality of these services for populations displaced by conflict.

This study reports baseline findings from a pre-post intervention study (examining the impact of more intensive health worker training and commodities for a package of newborn interventions) to describe the state of newborn facility-level care in displaced person camps in South Sudan. Specifically, the study will 1) describe the readiness of facilities to provide intrapartum and postnatal services, 2) assess midwife time-use during a work day, 3) document intrapartum and postnatal practices at the facility level, and 4) characterize maternal knowledge of newborn danger signs.

## Methods

### Design and setting

A cross-sectional descriptive design was employed using health facility assessments, direct observation of midwife time-use, clinical observation of the labor and delivery period, and exit interviews with recently delivered mothers. The study population consisted of mother-newborn pairs who sought services and birth attendants who provided delivery services between April and June 2016 in five health facilities operated by International Medical Corps (IMC) in displacement camps located in South Sudan: Maban (Gendrassa and Kaya), Juba, and Malakal. Maban hosts refugee camps, whereas Juba and Malakal have internally displaced person (IDP) camps. One hospital and four PHCCs that conduct the majority of deliveries in the camps agreed to participate in the study. The hospital, located in Juba, operated 24/7 with skilled attendants, an in-patient department and comprehensive emergency obstetric care, while the PHCCs have midwives and traditional birth attendants (TBA) conducting deliveries.

### Sampling and recruitment

Health facilities in the camps were selected based on: (1) provision of delivery care, (2) delivery of at least 20 births per month, and (3) management by IMC. The sample size for these current analyses of baseline data was based on power calculations conducted to ensure that the planned pre-post evaluation would be sufficiently powered to detect changes as a result of the intervention components. Male and female midwives, aged 18 and older, who were working a daytime shift on the date of data collection at one of the study facilities were eligible for time-use observation. A midwife was observed every ten minutes over an eight-hour shift for a four-day period at each facility to reach the necessary sample size. We estimated that midwives spend about 40 min of an eight-hour working day on postnatal care. To detect a 5% difference post-intervention in the proportion of time spent on postnatal care (e.g. 8% to 13%) with an alpha of 0.05, 0.8 power and 5% non-response rate, we required a total sample size of 620 observations at baseline and end line.

Women, aged 15 and older, arriving at a study facility for delivery during the 9-week enrollment period were eligible for recruitment for observation of the labour and delivery period and an exit interview. Similarly, the sample size calculated to see a change in baseline was 185 deliveries based on a two-sample comparison of the primary outcome—proportion of newborns that receive the components of essential newborn care (Table 1). This assumed a 50% baseline prevalence of the primary outcome, a conservative estimate due to a lack of data from similar settings, and a 15% detectable difference with an alpha of 0.05 and power of 0.8. The sample size accounted for a non-response rate of 8% based on a 3% stillbirth rate and 5% referral for complications. The sample was doubled to allow for analyses stratified by facility type: hospital and PHCC. Women delivering a stillbirth were subsequently excluded from analysis of observation data. Recently delivered mothers who participated in a clinical observation and did not have a stillbirth or neonatal death were asked to participate in an exit interview.

**Table 1:** Components of essential newborn care measured in the clinical observations

Thermal care: dried immediately after birth, wrapped/covered with dry towel, skin-to-skin contact, and delayed bathing while in facility Infection prevention: handwashing prior to delivery by health worker, eye care with tetracycline, and dry cord care while in facility Feeding support: skin-to-skin contact, breastfeeding in the first hour, and exclusive breastfeeding while in facility Postnatal monitoring: weight taken using a scale and temperature taken using a thermometer	

### Data collection

Locally-based non-clinicians were hired as research assistants and trained jointly to standardize data collection procedures across the health facilities. The three research teams consisted of three to five research assistants and one site supervisor. A ten-day training was conducted by study co-investigators to orient the teams to newborn clinical practices, research ethics and procedures, and the study instruments. Researchers practiced the interview aloud in both Arabic and Nuer languages to ensure the translation was standard between researchers. To improve reliability, a two-day pilot session was held to standardize observations between researchers, with one day in the classroom observing videos as a group and one day in a facility observing live births with researchers from the same site. Site supervisors received additional training to conduct the health facility assessment.

Data collection occurred at the five study facilities, starting with a health facility assessment. In April 2016, study health facilities were assessed for readiness to provide intrapartum and postnatal services using a structured questionnaire and checklist on the mobile device. The site supervisor interviewed the midwife in-charge about the availability of skilled birth attendants, running water, and electricity over the past month. Medical equipment, supplies and drugs for intrapartum and postnatal care were observed for availability in the delivery and postnatal care wards, and in relevant cases for functionality or expiration. The checklist for medical commodities was based on the recommended newborn supplies outlined in the *Newborn Health in Humanitarian Settings Field Guide*.

Following the assessment, we conducted the time-use observations. At each facility, for a four-day period, one midwife was randomly selected each day for time-use observation. Every ten minutes over an eight-hour period, the researcher recorded the main activity in which the midwife was engaged using a structured checklist; activities were categorized into eighteen general tasks for midwives such as postnatal care, intrapartum care, antenatal care (ANC), documentation, supervision, cleaning, and other tasks. Patient census data in the maternity unit was noted on every observation day. Informed consent was attained from each midwife.

For clinical observations, one researcher was assigned per shift to ensure continuous coverage during facility operating hours. Data collectors rotated every eight to twelve-hour shift, depending on the security guidance in each site. Each mother-newborn pair was observed for at least one and no more than 6 h before starting a new observation. The observation was documented using a structured checklist, based on the Maternal and Child Health Integrated Program (MCHIP) Quality of Care Surveys [15]. We observed the following clinical practices: (i) partograph use, (ii) preparation for delivery, (iii) immediate postnatal care, (iv) bag and mask resuscitation for non-breathing babies, and (v) kangaroo mother care (KMC) for low birth weight babies. Additional information on the outcome of the mother and newborn, gravidity, parity and birth weight was extracted from clinical records. Following observation and before the mother was discharged, researchers administered an exit interview using a structured questionnaire (in either Arabic or Nuer, according to the mother’s preference). Information was gathered on the mother’s demographic characteristics and her knowledge of six newborn danger signs without prompting response options. Verbal consent was obtained for both clinical observations and exit interviews from all participating mothers.

### Analysis

Data were entered on Android tablets using Magpi mobile data software (DataDyne Group LLC, Version 3.2.2). Data were monitored daily for quality checks and uploaded to an online server by site supervisors, reviewed for consistency, and exported to Stata (StataCorp LP, Version 13.1) for analysis. Descriptive analyses were performed to explore frequencies, means and interval estimation. Missing and “don’t know” responses were analyzed as missing and excluded from analysis. The activities measured in the time-use observations were classified into three categories: contact time for newborn care (by antenatal, delivery and postnatal care), non-contact productive time (i.e. documentation, meetings, training, supervision and cleaning) and non-productive time (i.e. waiting for patients, break time and socializing). Proportion of the total amount of observed time spent on each activity was estimated and a regression analysis was conducted to estimate associations with facility type. In the clinical observations, we conducted descriptive analyses of up to 65 practices for partograph use, delivery preparation, immediate postnatal care, neonatal resuscitation, and KMC. The primary outcome was defined by the proportion of newborns who received the essential components of immediate postnatal care: (i) thermal care, (ii) infection prevention, (iii) feeding support, and (iv) postnatal monitoring; individual sub-items contributing to these composite components are shown in Table 1. Partograph use was not analyzed among women who were admitted immediately to the labor room because they were assumed to be in the second stage of labour. For the exit interviews, the pre-defined response options for knowledge of newborn danger signs were entered as a binary (yes/no) variable. One point was assigned for each answer that was coded as ‘yes’, giving a maximum score of six. The overall knowledge score was categorized as ‘poor’ (zero to one danger signs), ‘moderate’ (two to three), and ‘adequate’ (four to six). Mother’s age, parity and years living in the camp were measured as a continuous variable and categorized as a binary variable for analysis. A chi-squared statistic was used to examine differences in newborn care practices by type of health worker and facility. Multiple logistic regression, in a stepwise backward manner with a 0.05 cutoff, was used to identify factors associated with newborn practices and maternal knowledge including maternal, newborn and health worker characteristics. In each step, variables that are least significant are removed until all variables in the regression model have a *p*-value of less than or equal to 0.05. Associations were examined by relative risks (RR), using a modified Poisson approach with a robust variance estimator, and 95% confidence intervals [16].

## Results

### Overview of participants

Among a total of 357 mothers recruited for observation across the hospital and PHCC strata, one refused to participate and 13 were excluded due to stillbirth or referral to another facility prior to delivery, leaving 343 mother-newborn observations for inclusion in the analysis. Less than 2% of the mothers who participated in an observation did not complete an exit interview before being discharged from the health facility.

Among the mother-newborn observations, 44.6% were from the hospital and 55.4% were from the four PHCCs (Table 2). Over two-thirds of observations had more than one health worker assisting with the delivery, although 25.4% were delivered by only TBAs. Maternal age ranged from 15 to 37 years, with a mean of 24.5 years (SD = 4.7). The proportion of mothers who received a visit from a community health worker (CHW) during pregnancy was 60% higher in the PHCCs than in the hospital (80.5% and 20.3%, respectively), while most mothers across all facilities attended at least one ANC visit (98.2%). Covariates with missing data resulted mainly from missing data in participants’ clinical records.

**Table 2:** Characteristics of observations among mother-newborn pairs by level of care, April–June 2016^*a*^

Variable^*b*^	Hospital		PHCC		All facilities	
	*n* = 153		*n* = 190		*N* = 343	
	No.	(%)	No.	(%)	No.	(%)
Type of health worker at delivery^*c*^						
Skilled birth attendant	153	(100.0)	103	(54.2)	256	(74.6)
Traditional birth attendant	0	(0.0)	87	(45.8)	87	(25.4)
Sex of health worker at delivery						
Male	79	(51.6)	18	(9.5)	97	(28.3)
Female	74	(48.4)	172	(90.5)	246	(71.7)
Mean birth weight, kg	3.0 ± 0.5		3.1 ± 0.6		3.1 ± 0.6	
Maternal gravidity						
1	24	(15.7)	29	(16.6)	53	(16.2)
2–4	88	(57.5)	85	(48.6)	173	(52.7)
≥ 5	41	(26.8)	61	(34.9)	102	(31.1)
Maternal education						
No school	115	(75.2)	135	(72.6)	250	(73.8)
Primary	30	(19.6)	39	(93.6)	69	(20.4)
Secondary or higher	6	(3.9)	10	(5.4)	16	(4.7)
Maternal employment						
Unemployed	152	(99.4)	168	(90.8)	320	(94.7)
Employed	1	(0.7)	17	(9.2)	18	(5.4)

^a^Values are given as number(percentage) and mean ± standard deviation

^b^Missing data for these variables was as follows: type of health worker, 0 (0.0%); sex of health worker, 0 (0.0%); birth weight, 35 (10.2%); gravidity, 15 (4.37%); education, 8 (2.3%); employment, 5 (1.5%)

^c^Skilled birth attendant defined as having a degree or certificate in midwifery

For the time-use observations, a total of 1116 observations were made of thirteen midwives at the five health facilities, with 16.9% of observations at the hospital and 83.1% at the PHCCs. Across the five facilities, 67.6% of the observations were among female midwives. The median age represented among the observations was 28 years (IQR: 26–29), with the majority receiving their midwifery degree in 2015 (63.8%). The observed midwives had an average daily caseload of 9.3 (SD = 8.2) consultations, 1.6 (SD = 1.7) deliveries, and 2.6 (SD = 4.1) postnatal care visits.

### Facility readiness to provide intrapartum and postnatal services

Overall, the majority of the five facilities had electricity (3 of the 5) and running water (5 of the 5) during the past month. The two PHCCs that had no electricity reported at least 15 to 30 days without electricity or a generator in the past month. Only two of the five facilities had skilled birth attendants (SBAs) working during all operating hours. The remaining three facilities had skilled attendants primarily in the daytime hours.

Readiness to deliver basic obstetric care was optimal at the hospital; however, none of the critical services (i.e. parenteral administration of oxytocin, assisted vaginal delivery, manual removal of retained products, or neonatal resuscitation) was found to be universally available across all PHCCs. All facilities were found to be lacking the recommended items to provide intrapartum and postnatal care for newborns (Table 3). The hospital, functioning at the referral level, had 20 of the 37 recommended medicines and equipment for newborn care service delivery and the PHCCs had on average 9 out of 25. Newborn-sized bag and mask, a thermometer and tetracycline eye ointment were the most commonly available newborn supplies across facilities. Although all facilities had a neonatal-sized bag and mask, none had a resuscitation table available in their delivery or postnatal area. Similarly, infant weighing scales were available but there were no KMC wraps for low birth weight babies. At the hospital level, the unavailable supplies were newborn clothing, wrap for KMC, neonatal-sized catheter, pediatric infusion set, pediatric pulse oximeter sensor, infant feeding tubes, oral phenobarbitone, amoxicillin (suspension powder), cefotaxime sodium and caffeine.

**Table 3:** Availability of medicine and equipment for newborn health service delivery in study facilities (*N* = 5)

Present in five facilities	Not present in five facilities
*Prematurity*	
Thermometer	Newborn clothing (cap, socks, romper)
Infant weighing scale	KMC wrap
	Feeding cup
	Nasogastric feeding tubes, for neonates
	Blood glucose meter
	Vitamin K-1 1 mg/ml
	Dextrose 10%, intravenous
	Phenobarbitone 54 mg/ml, oral
*Perinatal asphyxia*	
Newborn bag and mask	Doppler
	Resuscitation table
	Suction device
*Infection prevention and management*	
Tetracycline hydrochloride 1% eye ointment	Acyclovir 5% cream, 5 g
	Scalp vein infusion set, for neonates
	Ampicillin 500 mg; Benzylpencillin sodium 5 M I.U.; Gentamicin sulphate 40 mg/ml; Amoxicillin 125 mg/5 ml powder for suspension; Benzathine penicillin G 1.2 M I.U.; Artesunate 60 mg
*Advanced care (hospital only)*	
Oxygen concentrator	Pulse oximetry sensor, for neonates
Calcium gluconate 100 mg/ml	Caffeine 12.5 mg/ml
Ringer lactate/Hartmann’s solution, intravenous	Sodium bicarbonate 8.4%
Diazepam 5 mg/ml	Cefotaxime sodium 1 g
Procaine penicillin 1 M I.U.	Catheter I.V., with wings 24 G
Cloxacillin 250 mg	
Ceftriaxone 250 mg	

In a routine workday, the proportion of midwives’ time spent on patient care was 40.2% and the remaining time (59.8%) was spent in non-contact. Of the time spent on non-contact activities, slightly more than half was non-productive (51.9%); productive time (48.1%) was spent on cleaning, documentation, supervision and formal meetings. The main activity observed within patient care was ANC (53.2%) followed by delivery care (31.4%). Of the total observed time, 6.2% of midwives’ time was spent on postnatal care or about 30 min of an eight-hour work day. There was a statistically significant difference in the proportion of time spent on ANC (RR = 1.83, 95% CI: 1.23 to 2.70) and delivery care (RR = 0.53, 95% CI: 0.38 to 0.74) among the midwives observed in the PHCCs, relative to those observed in the hospital.

### Delivery and immediate postnatal practices

For the mother-newborn observations, 64.4% of mothers were admitted to the facility before second stage of labour, while others went directly to the labour room upon arrival. More than half of the mothers (57.0%) who arrived before the second stage of labour had a partograph used to monitor their fetal heart rate, with the majority (91.3%) having their fetal heart rate monitored every 30 min. The practice of partograph use was less common for mothers who delivered in a PHCC (25.7%) than a hospital (85.3%); difference: 59.6% (95% CI: 48.1% to 70.2%).

During the delivery period, all observed newborns were dried immediately with a towel and most received essential practices for newborn care (Table 4). Facilities indicated that the routine practice was to sterilize cord-cutting supplies, but we were unable to measure an actual observation of sterility. In the first hour after birth, a significantly higher proportion of mothers placed their newborn skin-to-skin in the hospital (88.9%) as compared to the PHCC (49.7%). Among those who provided skin-to-skin, the median time for skin-to-skin contact was also higher in the hospital (*Mdn* = 12 mins, IQR: 8–20) than the PHCC (*Mdn* = 1 min, IQR: 1–10). Nearly all newborns (95.9%) received tetracycline eye ointment for eye care in the first hour after birth, and a higher proportion received tetracycline by discharge. Similarly, a high proportion of mother-newborn pairs initiated breastfeeding, measured by observed latch, within the first hour in the hospital (98.7%) and PHCCs (97.3%).

**Table 4:** Immediate postnatal practices observed among mother-newborn pairs, by level of care

Outcome	Hospital		PHCC		
	Frequency	Percentage (95% CI)	Frequency	Percentage (95% CI)	*p*-value
Dried immediately with towel	153/153	100.0 (97.6–100)	187/187	100.0 (98.0–100)	0.366
Covered/wrapped immediately with dry towel	150/153	98.0 (94.4–99.6)	160/182	87.9 (82.3–92.3)	0.000
Tied or clamped cord after one minute	145/148	98.0 (94.2–99.6)	173/183	94.5 (90.2–97.3)	0.109
Placed skin-to-skin within 1st hour	136/153	88.9 (82.8–93.4)	93/187	49.7 (42.4–57.1)	0.000
Initiated breastfeeding within 1st hour	151/153	98.7 (95.4–99.8)	183/188	97.3 (93.9–99.1)	0.381
Applied tetracycline eye ointment	147/151	97.4 (93.4–99.3)	183/188	97.3 (93.9–99.1)	0.291
Weight taken using a scale	153/153	100 (97.6–100)	161/187	86.1 (80.3–90.7)	0.000
Temperature taken using a thermometer	19/151	12.6 (7.7–19.0)	77/188	41.0 (33.9–48.3)	0.000
Dry cord care while in facility	152/152	100.0 (97.6–100)	118/122	96.7 (91.8–99.1)	0.025
Exclusive breastfeeding while in facility	153/153	100.0 (97.6–100)	115/122	94.3 (88.5–97.7)	0.003
Delayed bathing while in facility	145/150	96.7 (92.4–98.9)	116/121	95.9 (90.6–98.6)	0.729

*CI* confidence interval

Essential newborn care was measured as four different components, with each component consisting of the respective immediate postnatal practices (Table 5). Mother-newborn pairs in the PHCCs were significantly more likely to receive infection prevention (RR = 1.28, 95% CI: 1.11 to 1.47) and postnatal monitoring (RR = 3.17, 95% CI: 2.01 to 5.00) practices than mother-newborn pairs in the hospital, without adjusting for potential confounders. This was not found for thermal care, where in the PHCC mother-newborn pairs were 52% less likely to receive thermal care when compared to the hospital (95% CI: 42% to 60%). The likelihood of receiving feeding support was similarly lower for PHCC-born newborns (RR = 0.49, 95% CI: 0.40 to 0.58). Table 5 shows the results of the final multivariate logistic regression model, with the hospital as the reference value. All components of essential newborn care remained strongly associated with facility type even after controlling for potential confounders.

**Table 5:** Essential newborn care practices observed among mother-newborn pairs in the PHCC vs. hospital: univariate and multivariate analysis

Outcome	Total no. of mother-newborn pairs	No. of mother-newborn pairs receiving outcome	Percentage	Univariate	Multivariate^e^
				RR (95% CI)	RR (95% CI)
Thermal care^*a*^	323	202	62.5	0.48 (0.40–0.58)	0.38 (0.29–0.50)
Infection prevention^*b*^	270	202	74.8	1.28 (1.11–1.47)	1.26 (1.06–1.52)
Feeding support^*c*^	327	208	63.6	0.49 (0.40–0.58)	0.38 (0.29–0.50)
Postnatal monitoring^*d*^	339	94	27.7	3.17 (2.01–5.00)	4.07 (2.58–6.40)

*RR* Relative risk; *CI* Confidence interval

^a^Thermal care defined as receiving dried immediately after birth, wrapped/covered with dry towel, skin-to-skin contact, and delayed bathing while in facility

^b^Infection prevention defined as having health worker handwashing prior to delivery, eye care with tetracycline, and dry cord care while in facility

^c^Feeding support defined as receiving skin-to-skin contact, breastfeeding in the first hour, and exclusive breastfeeding while in facility

^d^Postnatal monitoring defined as having weight taken using a scale and temperature taken

^e^Adjusted for skilled birth attendant, sex of health worker and low birth weight

In the PHCCs, about 45.8% of mother-newborn pairs in the study were attended by only a TBA during delivery. There was a statistically significantly higher proportion that received postnatal monitoring (RR 1.59, 95% CI: 1.09 to 2.32) among mother-newborn pairs delivered by a SBA as compared to those delivered by a TBA. However, other essential newborn care components were not statistically different.

Preparedness for neonatal resuscitation was assessed using a binary variable generated from observing the availability of a suction device and newborn bag and mask. Of the 343 deliveries observed in the facilities, 73.2% of them had supplies prepared for neonatal resuscitation; differences between facilities were not statistically significant (*p* = 0.224). While the number of resuscitation cases is not large enough for statistical analysis, 30 of the 343 observed newborns (8.8%) were not breathing at birth. Among those 30 resuscitation cases, 26 newborns began breathing after the health worker(s) initiated suction, drying, and/or back stimulation. The ventilation mask was correctly positioned and resuscitation conducted on the four remaining newborns, but only two newborns started breathing after its use.

Twenty-six (7.6%) of the 343 newborns were considered to be low birth weight (<2500 g) by the health worker(s) using a weighing scale or estimation of weight with no weighing scale. Among those newborns who were considered low birth weight, none were placed immediately in the proper KMC position.^1^

### Maternal knowledge of newborn danger signs

A total of 339 recently delivered mothers were interviewed before they were discharged from the health facility: 153 in the hospital and 186 in the PHCC (Table 6). Mothers’ knowledge of danger signs was poor, with about one-fifth of mothers correctly identifying four or more signs (20.4%). Although 44.8% of mothers reported fever as a danger sign for newborns, only 18.0% recalled baby feeling cold to the touch as a potential sign of illness. A low proportion of mothers had knowledge of other newborn danger signs such as reduced activity (27.7%), difficulty breathing (28.9%), and convulsions (11.2%). However, a relatively higher proportion of mothers (41.0%) described not feeding well as a danger sign for newborns. The median number of dangers signs that were recalled was one (IQR: 0–3), while 47.8% of mothers recalled none of the six components.

**Table 6:** Knowledge of newborn danger signs among recently delivered mothers, where multiple responses were elicited^*a*^

Danger sign reported	Hospital *n* = 153		PHCC *n* = 186	
	No.	(%)	No.	(%)
Fever	45	(29.4)	107	(57.5)
Feeling cold	14	(9.2)	47	(25.3)
Reduced activity	36	(23.5)	58	(31.2)
Difficulty breathing	36	(23.5)	62	(33.3)
Not feeding well	33	(21.6)	106	(57.0)
Convulsions	5	(3.3)	33	(17.7)
Knowledge of all newborn danger signs	2	(1.3)	25	(13.4)

^a^Values are given as number(percentage)

Mother’s education, having a home visit by a CHW, and number of years living in a camp were associated with mother’s ability to recall newborn danger signs and were shown to be significant (Table 7). Only 16.4% of mothers with no education had adequate knowledge of newborn danger signs; whereas, 32.9% of mothers with at least primary education had adequate knowledge (*p* = 0.003). Regarding number of years living in a camp, 29.8% of mothers who lived in the camp for one year or less had adequate recall of newborn danger signs compared to 17.1% of mothers who lived in the camp for more than one year (*p* = 0.034). Additionally, 28.3% of mothers who had a home visit by a CHW had adequate knowledge but only 11.5% of mothers who did not have a home visit were able to adequately recall four to six danger signs (*p* = 0.001). There was no significant association between the level of maternal knowledge of danger signs and mother’s age (*p* = 0.525) or mother’s employment (*p* = 0.364). Nearly equal proportions of recently delivered mothers who were primiparae and multiparae had adequate knowledge of the danger signs (23.1% and 20.2%, respectively).

**Table 7:** Factors associated with maternal knowledge of newborn danger signs: univariate analysis^a^

Characteristic	Total	Level of knowledge^b^			*p*-value
		Poor No. (%)	Moderate No. (%)	Adequate No. (%)	
Mother’s age, yrs					
≤ 25	211	116 (55.0)	54 (25.6)	41 (19.4)	0.525
> 25	128	74 (57.8)	26 (20.3)	28 (21.9)	
Parity					
Primiparae	52	28 (53.9)	12 (23.1)	12 (23.1)	0.888
Multiparae	273	151 (55.3)	67 (24.5)	55 (20.2)	
Mother’s education					
No school	250	144 (57.6)	65 (26.0)	41 (16.4)	0.003
Primary school or higher	85	43 (50.6)	14 (16.5)	28 (32.9)	
Mother’s employment					
Unemployed	320	181 (56.6)	76 (23.8)	63 (19.7)	0.364
Employed	18	8 (44.4)	4 (22.2)	6 (33.3)	
Home visit by CHW					
No	157	100 (63.7)	39 (24.8)	18 (11.5)	0.001
Yes	180	88 (48.9)	41 (22.8)	51 (28.3)	
Displacement status					
IDP	208	117 (56.3)	49 (23.6)	42 (20.2)	0.994
Refugee	131	73 (55.7)	31 (23.7)	27 (20.6)	
Years living in camp					
One year or less	84	44 (52.4)	15 (17.9)	25 (29.8)	0.034
More than one year	251	144 (57.4)	64 (25.5)	43 (17.1)	

^a^Values are given as number (percentage)

^b^Poor knowledge defined as 0–1 danger signs; moderate as 2–3 danger signs; adequate as 4–6 danger signs

## Discussion

Health statistics among populations displaced by conflict are limited. Our study provides evidence on newborn health care in South Sudan, which is facing a protracted humanitarian crisis. By utilizing multiple data collection methods and sources, we explain key facets of service provision that influence intrapartum and postnatal care for newborns. First, service availability and readiness for newborn care was a challenge for health care organizations at the facility level. As in many humanitarian settings, we found disruptions in electricity, missing medical commodities for newborn care, and staffing challenges marked by limited skilled attendants. Secondly, midwives in our study facilities spent the majority of time on non-patient contact activities, while low cost, highly effective elements of postnatal care were still missing. Lastly, mothers who recently delivered in our study facilities had inadequate knowledge of newborn danger signs that can lead to a delay in care-seeking behaviours. These effects together highlight the potential negative impact on neonatal health service delivery in South Sudan during a protracted humanitarian crisis.

Although our study facilities are not representative of all non-governmental operations, it is indicative of the infrastructure, staffing and supply challenges faced by many health care organizations in South Sudan [17–21]. In the Malakal and Maban PHCCs, we found that electricity was not available during all operating hours and was supplemented by flashlights in the evening hours. Similarly, many supplies recommended for intrapartum and postantal care were unavailable at the hospital and PHCC level. Signal functions proposed for routine and emergency newborn care include reliable electricity, skilled providers and medical commodities to achieve quality maternal and newborn care [22]. Complications of preterm birth is the leading cause of death in children under five years of age globally and a large burden of disability [1]. Many of the gaps observed likely compromise the ability of staff to provide quality care for these small babies who are more vulnerable to infection, hypothermia, under-nutrition, and other illnesses.

Cost-effective, evidence-based newborn care at the health facility alone can prevent up to 1.3 million newborn deaths if we reach 90% coverage [9]. Essential newborn care practices that were observed at high levels (>90%) included immediately drying the newborn with a towel, tying or clamping the cord after one minute, and breastfeeding initiation within the first hour. A previous study on facility births in Ethiopia reported lower estimates for immediate drying, although similar levels were seen in eastern Uganda [23, 24]. We found that breastfeeding initiation was considerably higher in our study when comparing to other studies [23, 24]. Although we saw high coverage of certain newborn practices, gaps still existed in essential newborn care. Practices such as time spent in skin-to-skin contact and taking the newborn’s temperature were low. World Health Organization strongly recommends skin-to-skin care in the first hour of life for all babies in order to encourage breastfeeding and protect against hypothermia [25]. Similarly, the temperature of a newborn should be assessed at each postnatal check beginning on day one [25]. We observed that a significant amount of midwives’ time was spent on non-contact activities and less than 30 min per day on postnatal care. Interventions such as prolonged skin-to-skin contact, particularly kangaroo mother care, require behavior change and more counseling time with midwives. Similarly, a health check for the baby prior to discharge and counseling on healthy home behaviours is not being prioritized. By shifting the responsibilities and time management of staff, and engaging community outreach services, there are opportunities to improve the uptake of these essential practices.

Availability of a skilled birth attendant during the intrapartum period is estimated to reduce neonatal mortality by 25–40% [9]. However, it is estimated that globally 25% of deliveries occur without a skilled birth attendant [12]. Humanitarian contexts, such as South Sudan, face great challenges in human resources due to flight of skilled personnel and deterioration of training institutions, and in practice we find that care by an unskilled workforce is common [26, 27]. Notably, most of the midwives in our study were recent national graduates relocated to the field and their attendance in the facilities was contingent upon the security situation. TBAs, who received on-the-job training and supervision, provided delivery and postnatal care during periods of insecurity and referred complicated deliveries to the closest hospital. We found that TBAs were less likely than SBAs to provide components of essential newborn care such as postnatal monitoring, while other practices were not statistically different. This is likely due to challenges with literacy and numeracy among TBAs that inhibited from measuring and documenting the newborn’s weight and temperature. Given that TBAs in our study conducted about half of the deliveries in the PHCCs, consideration should be given to increase midwifery training institutes and improve access to skilled attendants.

Differences were seen in intrapartum and postnatal care at the hospital and PHCC level, even after adjusting for skilled birth attendance. Use of the partograph, an important intervention for preventing intrapartum complications, was found to be less common at the primary care level. One explanation may be that service provision by SBAs was discontinuous in the PHCCs; thus, deterring the initiation of the partograph by skilled personnel. Another explanation can be that many mothers delayed reaching the health facility and delivered before partograph initiation. Practices for newborn thermal care and feeding support, such as covering the baby with a dry towel and skin-to-skin contact immediately after delivery, were more common in the hospital than the PHCC level. Alternatively, taking the baby’s temperature, a practice for postnatal monitoring, was less common in the hospital. One reason for these differences may be the higher number of deliveries occurring in the hospital, leaving less time to monitor the newborn. These differences in essential newborn care practices may also be indicative of the health system bottlenecks that are specific to the context or intervention. A multi-country analysis found the greatest barriers to basic newborn care in Africa were the health workforce (e.g. staff number, supportive supervision and training quality) and service delivery (e.g. guideline adherence and health worker attitudes) [28].

Our study also found poor maternal knowledge of newborn dangers signs. Intervention packages that integrated health education on danger sign recognition have seen improvements in care-seeking behaviors among mothers for their newborns [29]. However, almost half of the mothers who participated in our study could not recall any danger signs for newborns. Fever was the most commonly reported danger sign followed by not feeding well, difficulty breathing, reduced activity and feeling cold. Convulsing was the least commonly known danger sign. Similar findings were reported in rural Uganda where fever (20.0%), difficulty in breathing (29.4%) and not feeding well (20.0%) were the most commonly recognized danger signs, with convulsions (4.8%) and coldness (0.3%) as the lowest [30]. In Kenya, a similar trend was found but a greater proportion of mother’s recalled each danger sign [31]. One potential explanation for this difference is lower maternal education in the Uganda and South Sudan studies, in comparison to Kenya [30, 31]. Duration of time living in a camp has not been previously studied in association with recognition of newborn danger signs. In our study, mothers who lived less than one year in the camp had more knowledge of danger signs. This may be due to various contextual factors and require further investigation to understand this disparity in knowledge. Visitation by a CHW was strongly associated with maternal knowledge of newborn danger signs, as seen in rural Pakistan and South Africa [32, 33]. Opportunities to reach caregivers with education on danger signs through CHWs or their ANC visit could have a positive impact on newborn health outcomes [29, 34].

### Limitations

This study provides a cross-sectional snapshot of the services available in a specific period, and may not be representative of services during an acute phase of the conflict. Additionally, the data are not representative of newborn health services provided by government facilities in South Sudan where bottlenecks that affect care are likely exacerbated. However, findings may represent services seen in other camp-based, NGO facilities that follow the Minimum Initial Service Package (MISP) for Reproductive Health in Crises, in or outside of South Sudan [35]. Limitations to our methods include the potential for the Hawthorne effect by using direct observation of health worker time-use and clinical practices; however, researchers were discrete about the measures being observed and became integrated in the workforce over a nine-week period. Clinical observation methods rely on researchers to use a standard approach to observing practices. Clean or sterile cord cutting was implemented using different procedures across the facilities, and researchers were unable to standardize their observation methods. Furthermore, our sample size was not estimated to measure newborn resuscitation or KMC, which limited our analysis of these practices. Additionally, the exit interviews excluded mothers with a stillbirth or newborn death who may have been less knowledgeable of danger signs.

## Conclusions

Addressing health service delivery in contexts affected by conflict is vital to reducing the global newborn mortality rate. Protracted humanitarian settings, such as South Sudan, need long-term resources to overcome the considerable hurdles for reaching every woman and every child as envisioned to achieve the Sustainable Development Goals. Soon after this baseline study concluded in Juba, the maternity ward in the hospital was shelled, a cholera outbreak began, and there was ethnic targeting of medical staff. These dynamic changes call for prepositioning appropriately sized medical supplies and working with skilled attendants to prioritize lifesaving newborn health interventions as part of maternal services. Gaps in intrapartum and postnatal care, particularly skilled care at birth, suggest a critical need to build the capacity of the existing health workforce while simultaneously increasing access to skilled deliveries. In addition, further research is required to understand care for small and sick newborns in these settings, particularly during acute crisis and among lower-skilled health workers. Our study focused on facility-based services in a camp setting where the skilled birth attendance rate is likely substantially higher than outside of the camp. Further research should be conducted to understand the state of newborn care outside of camps in South Sudan including community-based delivery and postnatal care.

## Abbreviations

ANC: Antenatal care
CHW: Community health worker
IDP: Internally displaced person
KMC: Kangaroo mother care
MMR: Maternal mortality ratio
NGO: Non-governmental organization
NMR: Neonatal mortality rate
PHCC: Primary health care center
SBA: Skilled birth attendant
TBA: Traditional birth attendant

## Footnotes

1 KMC positioning is defined as holding the baby in a vertical position with the baby’s legs in frog position with skin-to-skin contact using a secured wrapper.
